# How Do Allied Health Professionals Construe the Role of the Remote Workforce? New Insight into Their Recruitment and Retention

**DOI:** 10.1371/journal.pone.0167256

**Published:** 2016-12-01

**Authors:** Narelle Campbell, Diann S. Eley, Lindy McAllister

**Affiliations:** 1 School of Medicine, The University of Queensland, Brisbane, Queensland, Australia; 2 Faculty of Health Sciences, The University of Sydney, Lidcombe, New South Wales, Australia; University of Ottawa, CANADA

## Abstract

**Purpose:**

Allied health workforce recruitment and retention in remote areas is a global problem. Using case studies from the Australian allied health workforce, this paper adds new information by combining personality trait information with a detailed understanding of how the cases construe the demands of remote work, which may be useful in addressing this problem.

**Methods:**

Four cases (two urban, two remote) are presented from a mixed methods study (n = 562), which used (1) the Temperament and Character Inventory to investigate personality traits of allied health professionals; and (2) repertory grid interviews to reveal quantitatively and qualitatively how the cases construed their *Ideal* work role compared with their *Current* and a *Remote* role. Cases also self-assessed their fit (‘suited’ or ‘not suited’) with remote.

**Findings:**

Differences in the way cases construed their fit with remote work was related to prior experience. However all were satisfied with their work, perceiving their *Current* role as similar to their *Ideal*. All saw remote work as requiring generalist expertise and a reliance on relationships. Personality traits, especially Novelty Seeking and Harm Avoidance, fit with how allied health professionals perceived their role.

**Conclusions:**

The combination of two distinct lines of investigation, illustrates what more can be revealed about allied health professional’s career choices by taking into account the fit or lack of fit between their personality tendencies, their construing of remote work and their life circumstances. Understanding the combined influence of perceptions and traits on an individual toward or away from remote work may enhance recruitment and retention internationally.

## Introduction

Recruitment and retention of the health workforce, including allied health allied health professionals, to rural and remote areas is a global problem [1–3]. Reduced access to health services disadvantages rural and remote residents and results in poorer health outcomes compared with residents of urban areas [4,5]. In Australia, similar to many parts of Canada and the United States of America (USA), remote regions are characterized by vast distances between large population centers, harsh geography and climate, small and dispersed populations, and populations that are more likely to be Indigenous. Health services in remote areas are usually primary care based with allied health services often provided from a regional center and travelling out to more remote communities.

Both internationally and within Australia, allied health professionals play a vital role in health care. The term ‘allied health’ represents a diverse group of tertiary-trained professions, each making a specialized contribution to the diagnosis, management and prevention of both chronic and acute health conditions [6]. Examples of core allied health professions include physiotherapy, occupational therapy, speech-language pathology, social work and dietetics.

Given the importance of allied health professionals in health care, maldistribution of the workforce is an important problem to address. In developed countries such as Australia, the USA and Canada, where stable and comprehensive health training and resources exist, investment in research, policy and programs designed to address the health workforce maldistribution have not been able to entirely solve the problem [3,7,8,]. Notably recruitment and subsequent retention of health professionals into remote areas has been widely researched in the medical and nursing professions but less so in allied health [9].

An emerging body of research suggests that personality characteristics may provide a new approach to recruitment and retention of the remote and rural health workforce [10–13]. Personality has been previously linked with both career satisfaction [14] and choice [15]. Within medicine, personality has been shown to influence specialty choice [16–18] and geographic location of practice [12,19].

Specific investigation into personality in allied health professionals is scant although it is acknowledged as a potential source of helpful information for recruitment and retention of individuals who ‘fit’ with the work demands [20]. Qualitative studies have described characteristics such as adventurousness, resourcefulness and flexibility associated with retention in remote areas [21,22]. Only a few studies have examined the personality traits of specific allied health professions. These have suggested that the traits exhibited by the allied health professions differ from the general population [13,23], that there appears to be a match between the demands of the profession and the attributes of the individual [23–27] and that there may be differences between allied health professions [28].

In addition to a potential influence from personal traits, recruitment and retention may also be influenced by the perception allied health professionals hold about remote workplaces. Career decision-making, including career transition points such as new graduates seeking their first position, has been shown to be influenced by perceptions, or construing, about particular workplaces and the associated work requirements [29]. In considering a remote position, an allied health professional is likely to combine their personal sense of being suited to remote work, with their construing about both the benefits and the challenges. Challenges widely noted in remote regions include professional and personal isolation, large workloads, limited professional development support and career options, and under-valuing of the allied health role; whereas the benefits included lifestyle, community connectedness, and the diversity of work [30].

This paper will add new information to the problem of recruitment and retention of allied health professionals to remote areas by using case studies to present a unique combination of data from a mixed methods design study. The data includes a validated personality measure and structured interviews (repertory grid interviews), to answer the two-part research question: How do allied health professionals construe themselves and others in relation to work success, particularly in light of their self-assessed ‘fit’ with the remote work environment; and What personality traits are associated with their construing of this fit?

### Using a personality measure to understand remote success

The Temperament and Character Inventory-Revised 140 (TCI) [31], based on Cloninger’s bio-psychosocial model of personality, provides a comprehensive assessment of the seven basic dimensions of personality. It measures four temperament traits considered to have a biological genetic basis and be mildly heritable; and three character traits considered modifiable or responsive to environmental and developmental influences. Table 1 outlines these traits with descriptors of low and high levels of the traits. The model suggests that it is the unique combination of trait levels that contributes to the expression of personality in an individual and that the interaction between the developmental nature of the character traits and the more stable temperament traits may result in personal trait differences over time.

**Table 1:** Temperament and Character Trait descriptors*.

**Temperament Dimensions**	Description of dimension with **Low and High levels observed as:**		
Novelty Seeking	Exploratory activity in response to novelty, impulsiveness, and extravagance		
	indifferent, reflective frugal, detached, orderly, regimented	↔	exploratory, curious, impulsive, disorderly, extravagant, enthusiastic, seeks challenge
Harm Avoidance	Pessimistic worry in anticipation of problems, fear of uncertainty, shyness with strangers, and rapid fatigability		
	relaxed, optimistic, bold, confident, outgoing, vigorous, opinionated, decisive	↔	worrying, pessimistic, fearful, doubtful, shy, fatigable, indecisive
Reward Dependence	Social reward observed as sentimentality, social sensitivity, attachment, and dependence on approval by others		
	practical, cold, withdrawn, detached, independent, not influenced by others, socially insensitive	↔	sentimental, warm, dedicated, attached, dependent, needs to please, seeks approval from others
Persistence	Behavior despite frustration, fatigue and reinforcement; observed as industriousness, determination and perfectionism		
	inactive, indolent, gives up easily, un-ambitious underachiever, quitting, pragmatist	↔	industrious, diligent, hard-working, ambitious, overachiever, perseverant, perfectionist, determined
**Character Dimensions**	Description of dimension with **Low and High levels observed as:**		
Self Directedness	Extent to which an individual is responsible, reliable, resourceful, goal-oriented and self-confident		
	blaming, unreliable, purposeless, inert, ineffective, habits congruent with short-term goals	↔	responsible, reliable, purposeful, resourceful, effective habits congruent with long-term goals
Cooperativeness	Extent to which individuals are cooperative, tolerant, empathic and principled		
	socially intolerant, critical, unhelpful, revengeful, destructive, opportunistic	↔	socially tolerant, empathic, helpful, compassionate, constructive, ethical, principled
Self Transcendence	Extent to which individuals conceive themselves in relation to the universe as a whole. Observed as spirituality, practicality, materialism and modesty		
	impatient, unimaginative, proud, lack of humility, materialistic, practical	↔	wise, patient, creative, imaginative, self-effacing, united with universe, modest, humble, spiritual

*Adapted from Cloninger et al [32].

The TCI has been widely validated, both internationally [33,34], and in Australia [19,35]. It shares variance with other personality measures [36–38] and has been used to investigate personality in an array of health professions including anesthetists and physicians [39], nurses [40], rural doctors [11] and medical students [18,41]. Recent findings using the TCI with Australian allied health professionals suggested that allied health professionals with remote experience were higher in Novelty Seeking and lower in Harm Avoidance than the general population [13].

### Using repertory grid interviews to understand remote success

Repertory grid technique is a structured interview process and the key data collection tool for personal construct psychology [42,43]. Personal construct psychology emphasizes the individual’s beliefs and perceptions about their world. The beliefs held by an individual are personal to them, can be revised or discarded, and assist them in making sense of life. In personal construct psychology these beliefs are called ***constructs***.

Constructs are formed around specific topics of pertinence. Examining one’s constructs for their usefulness can result in productive or counter-productive changes in thinking for individuals. In addition, constructs are bipolar, meaning that if an individual believes one thing, they do not simultaneously believe the opposite. For example, a belief that working in a remote location undermines career advancement may inhibit an individual from seeking a remote position. In comparison, coming to believe that career advancement could be accelerated by the broad opportunities available in remote positions may motivate an individual to seek remote work. Therefore understanding the constructs held by allied health professionals in relation to their own work, and that of the remote allied health professional, could provide insight into the recruitment and retention potential of individuals. Examining the constructs for commonalities could assist in development of appropriate recruitment and retention policies for remote areas. The purpose of the repertory grid interview then, is to reveal the constructs.

This paper uses a combination of personality trait and repertory grid data, presented through case studies that will illustrate the recruitment and/or retention potential of allied health professionals to geographically remote workplaces. Each case study will present a picture of personality characteristics likely to influence the individual’s perceived fit with the demands of the remote work environment, and demonstrate how their construing or perceptions about their personal fit within the remote environment may influence their recruitment and retention potential.

## Method

### Ethics approval and consent processes

Ethics approval was obtained through The University of Queensland Medical Research Ethics Committee (Approval no. 20100000872). The consent process approved by the Ethics committee included participants receiving written informed consent documents which could be signed or emailed back to the researchers. All participants provided informed written consent.

### Case sampling

The four case studies were selected from the sample of 562 allied health professionals who had participated in a multi-stage mixed methods investigation into personality and motivation characteristics of allied health professionals. (See [13,25] for further details.) The investigation included a survey containing demographic questions and a personality inventory, the TCI; followed by repertory grid interviews with a smaller purposive subset (n = 34) of the sample that stratified for profession, sex, level of experience, current work location and their self-assessed fit with remote work (‘I think I am suited’; ‘I think I am not suited’). The TCI results of the subset were not significantly different from the whole sample (n = 562).

For this paper, four case studies from the repertory grid sample were purposively selected for in-depth analysis. The rationale for selection included mirroring the gender ratio and range of professions in the sample of 562, and contrasting between the individuals on four criteria:

- Level of experience (less than three years for novice),
- Self-assessment as suited (or not) to remote work,
- Current work location (remote/urban),
- Intention to work in remote if not already located there.

These four individuals, shown in Table 2 below, therefore share commonalities and differences which will enable an understanding of their recruitment and/or retention potential and which might be illuminating in regard to the broader allied health professional workforce.

**Table 2:** Key selection criteria for case study participants.

	Less than 3 years professional experience	Self-assessed as suited to remote	Currently working in remote	Intended to work in remote
**Ben**	√	√	x	√
**Teagan**	√	x	x	x
**Kylie**	√	√	√	√
**Nicole**	x	√	√	√

The remainder of the paper will refer to these cases as ‘participants’.

### The survey

The survey contained the 140 item TCI [32] which uses a five point Likert scale (1 = absolutely false; 5 = absolutely true). Internal reliability (Cronbach Alpha) of each trait ranged from 0.86 to 0.89 for the character and 0.69 to 0.91 for the temperament traits.

Demographic variables collected were: sex, age, profession, background (remote/rural/urban), family characteristics, years since graduation, work experience (remote/rural/urban), and self-assessed fit with working in a remote environment.

### The repertory grid interview

A repertory grid interview has several steps. It first aims to reveal the ***constructs*** or beliefs held by the person being interviewed about the topic. It then requires the person being interviewed to systematically compare and ***rate*** the extent to which each construct applies to specific ***elements***. Elements in this study were work roles or titles that could be held by the allied health professional. Importantly, constructs are bipolar, thus each element can be rated as to how similar or dissimilar it is to one or other pole of the construct.

**Fig 1:**
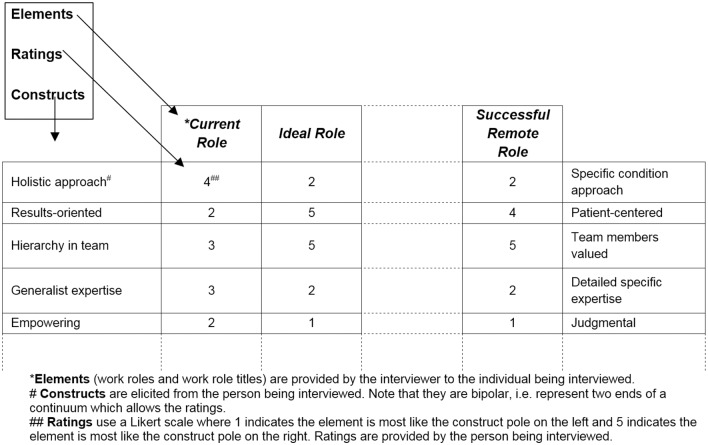
Illustrative sample of a repertory grid interview form.

The interview is recorded in real time on a grid which contains the elements, constructs and ratings. Fig 1 shows a simplified example of a completed repertory grid showing how the data elicited is both qualitative, through the constructs; and quantitative, through the ratings of the elements on each construct [44].

The elements selected for the repertory grid interview were allied health professional work roles/titles, including *Current* work position, *Ideal* position, and a *Successful Remote* professional. The *Ideal* element acted as the standard against which the other elements (work roles) were compared in terms of the participants preferred work role characteristics [45].

Following standard repertory grid practice [44], constructs were elicited using triadic presentation. This means the participant compared three elements at a time and articulated how two were the same as each other but different from the third. Specifically the question guiding them was ‘How are two of these work roles alike and different from the third in relation to personality or motivation factors that contribute to success at work?’ Participants rated each construct across all elements immediately after developing it.

### Analysis

The survey results (TCI scores and demographic data) were analyzed using IBM SPSS Statistics 19. (For a full description of the statistical analysis and results of the entire sample see [13, 25].)

Repertory grid interview data was analyzed with Idiogrid v2.4 [46], purpose-built repertory grid software. Quantitative analysis used the ratings to calculate the double-scaled Euclidean distances between elements, specifically between the *Ideal*, *Current* and *Successful Remote* for this paper. As a standardized measure of the similarity between pairs of elements, double-scaled Euclidean distance allows comparison between elements and across participants [47]. The results fall between 0 and 1, where 0 represents perfect similarity between the elements and 1 represents maximum dissimilarity. This means the distance between elements construed as alike will be closer to 0 compared with distances between elements construed as unlike being closer to 1.

Qualitative analysis of the constructs followed a standard repertory grid coding procedure [44] and resulted in a thematic analysis of the constructs. The first construct was examined for meaning and assigned a code. Subsequent constructs were compared for their fit with previously developed code(s) and either assigned to an existing code or a new code was created. The primary author undertook the initial coding and thematic analysis, then the other authors independently analyzed approximately 10% of the constructs. Differences in coding and the meaning of codes was discussed and negotiated until agreement was reached over three iterative cycles.

## Results

The key characteristics of the participants in this paper are shown in Table 3. To protect anonymity, the profession (speech-language pathology, dietetics, occupational therapy and social work) of the participant is not included in the table. The participants typified the female to male ratio common in allied health. The table also shows that each participant represented a different combination of experience, self-assessed fit with remote, and current work location.

**Table 3:** Key characteristics of case study participants.

Dimension	Ben*	Teagan*	Kylie*	Nicole*
**Professional experience**	<3 years (novice)	<3 years (novice)	<3 years (novice)	>5 years (experienced)
**Self-assessed fit with remote**	Suited	Not suited	Suited	Suited
**Current work location**	Urban	Urban	Remote	Remote
**Background**	Urban	Rural	Rural	Urban
**Partnered (background)**	Yes (rural)	Yes (rural)	No	Yes (rural)
**Dependents**	No	No	No	Yes
**TCI levels (population rank**^#^^)^	Note1	Note 2	Note 3	Note 4
Novelty Seeking	51 (average)	57 (high)	61 (very high)	54 (average)
Harm Avoidance	44 (low)	65 (very high)	54 (average)	39 (very low)
Reward Dependence	71 (very high)	77 (very high)	60 (high)	85 (very high)
Persistence	79 (very high)	82 (very high)	67 (very high)	84 (very high)
Self Directedness	82 (very high)	73 (very high)	80 (very high)	84 (very high)
Cooperativeness	80 (very high)	85 (very high)	89 (very high)	85 (very high)
Self Transcendence	54 (average)	53 (average)	39 (very low)	56 (high)
^##^**Double-scaled Euclidean distance between elements:**				
*Ideal*:*Current*	0.46 (neither like nor unlike)	0.25 (like)	0.40 (neither like nor unlike)	0.13 (like)
*Ideal*:*Succcessful Remote*	0.14 (like)	0.65 (unlike)	0.20 (like)	0.13 (like)

*pseudonyms.

^#^ Population rank to percentile: Very Low 0–16.7%; Low = 17–33%; Average = 34–66.7%; High = 67–83.3%; Very High = 84–100%.

Note 1: Ben’s TCI trait levels within 1 SD of allied health professional sample TCI mean.

Note 2: Teagan’s TCI trait levels within 1 SD of allied health professional sample TCI mean except for Harm Avoidance.

Note 3: Kylie’s TCI trait levels within 1 SD of allied health professional sample except for Reward Dependence.

Note 4: Nicole’s TCI trait levels within 1 SD of allied health professional sample mean except for Harm Avoidance, Reward Dependence and Persistence.

^##^ Distance calculated using double scaled Euclidean distance between elements where 0 = perfect similarity and 1 = complete dissimilarity between the two elements being compared.

In Table 3, the participant’s TCI levels are presented for each of the seven traits. This level is then compared to the population rank (low through to high). Additionally the table includes a note comparing each participant’s trait levels with the mean and standard deviation of the levels of the whole sample of 562 allied health professionals previously reported [13,25]. Importantly, all four participants have average or higher levels of Novelty Seeking. Additionally, Harm Avoidance levels are average or lower in all participants, except Teagan, who has very high levels. All show high or very high levels of Reward Dependence, Persistence, Self Directedness and Cooperativeness.

### The significance of the distance between elements

**Fig 2:**
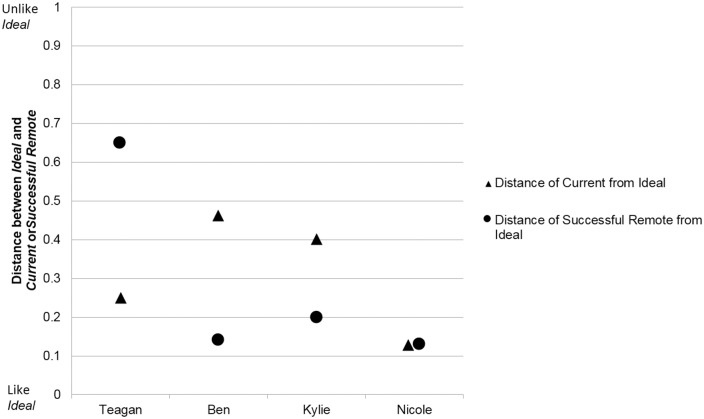
Distance for each participant from their *Ideal* element to their *Current* and *Successful Remote* elements.

Elements represent how each participant construed those work roles. The double-scaled Euclidean distances provide a comparison of the *Ideal* (representing the participant’s preferred work attributes) with two other roles, their *Current* role and the *Successful Remote*. Smaller distances indicate greater similarity between the roles. These distances are shown numerically in Table 3. Fig 2 shows the distances visually and compares the distances within and across participants. The *y* axis shows the distance between the participants *Ideal* and the other elements (either *Current* or *Successful Remote*).

As shown in Fig 2 all four participants had less than 0.5 distance between their *Ideal* role and their *Current* role, indicating that their *Current* was similar to their *Ideal*. In particular Nicole’s *Current* was very like her *Ideal* (distance of 0.13).

Three participants had very small distances between *Ideal* and *Successful Remote* indicating that they construed their *Ideal* as very similar to the *Successful Remote* role. In contrast, Teagan’s *Ideal* was quite unlike the *Successful Remote* (distance of 0.65).

### Construing about remote work: The significance of the constructs

Examination of the constructs developed by the four participants provided qualitative insight into the repertory grid data. The constructs showed how they perceived working in remote. The constructs characterizing *Successful Remote* fit into four major codes. These codes, shown in Table 4, were; Expertise, Value placed on the role, Relationships, and Career development. The table summarizes the construing of each participant about *Successful Remote* by showing the codes developed during analysis by the researchers in the left column. The constructs developed by each participant, but assigned to that code by the researcher, are in the following columns. Construing about *Ideal* is shown in brackets if it was very different to *Successful Remote*.

**Table 4:** Qualitative codes derived from constructs differentiating remote work.

Code	Constructs			
	Ben	Teagan	Kylie	Nicole
Expertise	Generalized knowledge*	Stimulating diverse work; Not specialized; (whereas *Ideal* = specialized knowledge and skills with some diversity)	Holistic understanding; generalist skills and knowledge (whereas *Ideal* = recognized for advanced generalist skills and knowledge)	Broad range of clients, skills and knowledge*
Value placed on role	Team understands and values all roles*	Not appreciated or making a difference; (whereas *Ideal* = role recognized and valued by colleagues)	Patients appreciative*	Community values role*
Relationships	Patient-centered; empathic*	Sense of belonging to community; Not anonymous; (whereas *Ideal* = anonymity and separation of personal and professional roles)	Client-centered, service provision more important than efficiency*	Holistic or family-centered; Community involvement personally and professional fulfilling; Trusted to do a good job*
Career development	#	Career development needs not recognized; Employment less stable; (whereas *Ideal* = stable employment and supported career development)	#	#

*the *Successful Remote* was like the *Ideal* for this construct.

^*#*^ the participant did not develop and rate a construct related to this code, i.e. the code was not relevant to the participant’s construing about remote work [44].

The constructs in Table 4 show qualitatively how Ben and Nicole construed their *Successful Remote*, and specifically that it was like their *Ideal*. Kylie construed her *Ideal* differently to *Successful Remote* only on the code of expertise where her *Ideal* received recognition for advanced skills. Note that Teagan’s construing of *Ideal* was different from *Successful Remote* for every code.

## Discussion

This paper has investigated how four allied health professionals, illustrative of the potential and existing remote allied health workforce, construed remote allied health work. Of note is that the approach taken, combining personality traits and self-assessed fit with remote work, is a unique way to consider the problem of recruitment and retention of the remote allied health workforce.

In terms of personality traits, our four participants were found to be within the range of the whole sample. However, the levels and combinations of certain traits suggest a potential influence on their construing of remote work. The temperament traits that may have particular importance to the participants construing about their fit with remote work are lowered Harm Avoidance (a calm and accepting approach to uncertainty), and elevated Novelty Seeking (an exploratory curious approach to novelty) [11]. These were combined with character traits showing high levels of Persistence (industrious and determination despite fatigue), Reward Dependence (warmth and social attachment) and Self Directedness (responsible and resourceful) all of which are known to contribute to coping in remote environments. This trait profile aligns with previously published literature on rural and remote health professionals [11,13,19,48].

The Euclidean distances calculated on the repertory grid data provide insight into two aspects that might influence successful recruitment and retention in remote. These are job satisfaction and identification with remote work.

Given that the *Ideal* element represented the participants’ preferred work characteristics, the distance between *Ideal* and *Current* for each participant is an indicator of job satisfaction [49]. In general, being satisfied at work could influence retention positively. The small distances (less than 0.5) shown in both Table 3 and Fig 2 suggest that all four participants were satisfied because they construed their *Current* position as more like (i.e. closer to) than unlike (i.e. further from) their *Ideal*. Ben, an urban novice, was the least satisfied, while Nicole’s distance of 0.13 showed the most similarity between her *Current* remote position and her *Ideal*, suggesting she is the most satisfied.

The distance between *Ideal* and *Successful Remote* compared participants’ preferred work characteristics with their construing about successfully working in remote. It thus suggests their identification (or not) with remote work. Neither Teagan nor Ben were working in remote. As seen in Fig 2, Ben’s small distance (0.14) between *Ideal* and *Successful Remote* suggested he identifies with remote work and therefore may be more receptive to working in remote. Teagan’s large distance (0.65) indicated reduced likelihood of her working in remote and that she construed *Successful Remote* very differently to her *Ideal* role. Both remote participants, Kylie and Nicole, had small distances between their *Ideal* and *Successful Remote* (0.2; 0.13 respectively) suggesting they identify with *Successful Remote* which may be a positive indicator for retention.

The literature notes that rural background and family/partner preferences influence recruitment and retention [19]. While our sample was too small to investigate an association between rural background or family factors (Table 3) with recruitment, both Nicole (urban background, experienced remote professional) and Teagan (rural background, no intention to work in rural/remote) demonstrate that these demographic factors are only one of a number of influences on recruitment to remote work.

Each participant will now be discussed in detail, unpacking their individual potential for the rural/remote workforce.

Ben, a novice urban allied health professional, provided a number of clues suggestive of potential for recruitment to remote. Firstly he self-assessed as being suited to remote, and although satisfied in his *Current* position, his *Ideal* was construed as like his *Successful Remote* (Fig 2), indicating that he identified with remote work and working in remote may increase his job satisfaction. The constructs, his construing of remote work, indicated his belief that remote work requires generalist expertise rather than specialist, and underscored the importance of the patient relationship and a team approach in enhancing quality patient care. Interestingly the absence of constructs related to career development opportunities suggest he was not concerned about career limitations in remote. His levels of certain personality traits appear to support his intention to relocate to remote. For example, his low Harm Avoidance combined with high Self Directedness indicate a low anxiety/high goal-achieving approach therefore potentially comfortable with moving away from known urban work contexts to more unknown and challenging remote situations.

Teagan, like Ben, was also a novice urban allied health professional, but appeared to have far less potential for recruitment to remote. The first indicator is the combination of her self-assessment as unsuited to remote work and her high job satisfaction in her *Current* urban role. Further there was considerable dissimilarity between her *Ideal* and *Successful Remote* (Fig 2) suggesting she did not identify with remote roles. While her construing of the remote work environment appeared realistic particularly regarding expectations of generalist expertise and loss of anonymity [30], her preference was for a work environment that endorsed specialist expertise and separated professional and personal roles. Construing that remote allied health professional roles were not appreciated, lacked career development opportunities and were unstable further decreases the possibility that she might consider a remote role. Compared to population norms, Teagan had a very high level of Harm Avoidance which may be associated with increased anxiety and difficulty in accepting the uncertainty inevitable in remote work. Her levels of Harm Avoidance, in combination with her construing of the remote work environment may be influencing Teagan’s decision to remain in urban.

Kylie, a remote novice with prior experience in both urban and rural areas, and a rural background, classified herself as suited to remote work. The relative closeness of her *Ideal*, *Current* and *Successful Remote* elements (Fig 2) suggested she was satisfied in remote roles and identified with the work of a remote allied health professional. The key constructs she developed mirrored the construing of the other participants about remote work such as ability to use a holistic approach in managing generalist caseloads and the importance of relationships. Of note is that despite high levels of Novelty Seeking (curiosity and desire for new challenges), Kylie reported that her *Current* role was the longest she has stayed in one allied health work role. Potentially the scope of work in her previous urban positions became mundane more quickly compared with the broad generalist expertise required in remote work. Her lower Reward Dependence suggests she is comfortable with her own company and less dependent on others for social stimulation which may fit well with a small remote community. Thus both her personal traits and her repertory grid results indicate she is well suited and potentially likely to be retained in remote areas.

Nicole, an experienced remote allied health professional described herself as suited and likely to remain in remote. The distances between her *Current*, *Ideal* and *Successful Remote* elements (Fig 2) were very small suggesting high job satisfaction and identification with the work of a remote allied health professional. Her constructs centered on her enjoyment of the generalist expertise required, and the value she placed on her close professional and personal relationship with the community. Furthermore her personality profile appears well aligned with her career decisions. She has very low Harm Avoidance indicating a tendency toward low anxiety which is conducive to an acceptance of the uncertainty associated with remote practice. High levels of Persistence may have contributed to her determination to work through challenges inherent in remote work and her very high Reward Dependence fits with the satisfaction and value she places on her professional and personal relationship with the community. These factors all support the likelihood of Nicole’s retention.

Overall these case studies revealed a number of insights about the individuals that might indicate remote workforce potential. These insights include positive self-assessed fit with remote, realistic construing of the remote environment, overall satisfaction with their current work position, and personality traits that appear to support success in remote, in particular lower Harm Avoidance and elevated Novelty Seeking. These findings are likely to be useful in thinking about remote allied health workforce recruitment and retention by employers and policy makers both within and outside Australia.

The findings also suggest possible actions to improve recruitment potential for individuals who might not initially consider remote work. Teagan illustrated this. Enabling Teagan to re-construe remote work and consider a career transition [29] to remote, would require practical policy changes to positively influence the remote work environment. These could include clear opportunities for career progression, increased value placed by consumers, other team members and the remote health system on the value of the allied health professional role, and support that alleviated her discomfort with holding dual professional and personal roles in a small community.

While this was an Australian study, application to American and Canadian contexts can be made given similarity with workforce shortages in rural and remote areas, training and certification processes to recognize allied health professionals, and health care based on western science. Policies that account for an individual’s perception of their fit with remote work, while acknowledging their personal traits and addressing the challenges represent an investment in the remote health workforce and thus might assist in better health outcomes for remote residents. Additionally, increased focus in training programs on the benefits and challenges of working in rural and remote could assist the development of allied health professionals who constitute a good fit with remote and who construe remote workplaces as providing satisfying and desirable employment.

### Limitations and future directions

Participants self-selected to this study so may potentially be biased in personality traits or attitudes towards remote work. It should also be noted that this paper is observational only and does not claim to infer any cause and effect. There is no suggestion of providing a comprehensive personality profile but rather to highlight the potential interplay of personal traits, work and life contexts and personal construing or perceptions of the work environment.

This study was limited to four case studies. Space did not permit the addition of participants covering all possible combinations of experience, self-assessed fit and identification with remote, job satisfaction and personal traits. Further investigation of all the participants who provided repertory grid interviews may deepen our understanding of the influence of these factors on recruitment and retention.

## Conclusion

Using a unique combination of quantitative and qualitative data, this paper has contributed new information pertinent to addressing the international issue of allied health workforce shortages in rural and remote areas. While a ‘one size fits all’ approach is unlikely to be successful, similarities in the challenges of the remote health workforce shortage are global. Much can be learned from collaborative sharing of strategies to improve remote recruitment and retention amongst the allied health professions [19].

This paper highlights the importance of looking at individual traits and perceptions that influence allied health professionals toward or away from remote work. It showcases successful recruitment and retention, and suggests that allied health professionals with a pre-disposition to remote work may exhibit personal traits helpful in managing the work environment.

This sample of allied health professionals were relatively homogenous in their personality profiles and represent characteristics that would be expected of health professionals. However, we must not forget that life circumstances, available opportunities and work environments, can be a strong influence in an individual’s career decisions. This work, combining two distinct lines of investigation, illustrates what more can be revealed about an allied health professional’s career choices by taking into account the fit or lack of fit between their personality tendencies and their construing of remote work and their life circumstances.

## Supporting Information

S1 File Case Study Repertory Grid Dataset. (DOCX)
