# Supplementary material for: How Do Allied Health Professionals Construe the Role of the Remote Workforce? New Insight into Their Recruitment and Retention
Source: PLoS One. 2016 Dec 1;11(12):e0167256. doi: 10.1371/journal.pone.0167256 (PMC5131943; doi:10.1371/journal.pone.0167256)
Supplement: S1 File — (DOCX) [file pone.0167256.s001.docx]

# Case study Repertory grid dataSet

## Repertory Grid B

| \| **Personal/motivation qualities that contribute to successful practice in the job**  **1** \| \| --- \| | **Myself as I am currently** | **Myself in my ideal job** | **Most successful remote practitioner** | **5** |
| --- | --- | --- | --- | --- | --- |
| **Construct (**similarity pole) | Ratings from 1 on the left to 5 on the right | | | **Construct** (contrast pole) |
| Holistic experience - role was broad and managed patient in different ways and over a period of time including after discharge and accounting for all of their conditions | 4 | 2 | 2 | Patient care is handed on to someone else, managing a very specific aspect of their care |
| Work with patients is results- orientated e.g. to set up a service, to give a prescription for a diet; driven by bureaucracy | 2 | 5 | 4 | Work with patients is patient-centred and around rapport, listening to the patients issues |
| Team relationships are dominated by medical doctors, a hierarchy where allied health professionals are not valued, communication is poor- notes not read, people not discuss issues | 3 | 5 | 5 | The team has a good understanding of other people's roles and where they are coming from with their recommendations, time is made available to communicate well, team members put in effort with communication including good listening skills |
| Range of work locations, varied patient presentations, wide cross-section of patients requires generalised knowledge | 3 | 2 | 2 | Similar patient groups, specific and unchangeable type of work and location requiring highly detailed, analytical and technical knowledge and expertise in an area |
| Empathy, empowering people and providing people with knowledge to motivate them | 2 | 1 | 1 | Telling people what they need to do without considering their background and reasons for not following recommendations; judging non-compliant patients |
| Motivated to work with economically and geographically disadvantaged people; people with hardships who don't necessarily have a lot of choice or knowledge - the service is needed vitally and often underfunding | 3 | 2 | 1 | Motivated to work in a job where people already have a lot of advantage e.g. sports dietetics; or cultural similarities; no breadth of difference across workload; focus of work is on people who have choice and knowledge- the service is a luxury; may have prestige and is often financially more viable |

## Repertory Grid T

| \| **Personal/motivation qualities that contribute to successful practice in the job**  **1** \| \| --- \| | **Myself as I am currently** | **Myself in my ideal job** | **Most successful remote practitioner** | **5** |
| --- | --- | --- | --- | --- | --- |
| **Construct (similarity pole)** | Ratings from 1 on the left to 5 on the right | | | **Construct (contrast pole)** |
| Stability of employment | 2 | 1 | 4 | Employment vulnerable to redundancy because of funding (project funding) |
| Freedom to be creative with your role | 1 | 1 | 4 | Position is structured with limited opportunities to be creative and innovative in doing things differently |
| Opportunity for effective interdisciplinary practice to provide holistic care; allows for collaborative problem-solving and support | 1 | 2 | 3 | Discipline-only based work; environment doesn't value strong teamwork |
| Specialist knowledge and competency | 2 | 1 | 3 | Less aware of role definitions, not specialised and needing to be directed by someone else |
| Value placed on leadership and taking/providing/supporting leadership opportunities | 2 | 1 | 2 | Set role/routine work that has to be done |
| Recognition and valued placed on the role by other colleagues and other disciplines | 3 | 1 | 4 | Your work is not appreciated and perception that it doesn't make a difference; your input are not appreciated |
| Workplace supports learning opportunities financially as well as using strategies such as exposure to diverse cases | 1 | 1 | 5 | Needs of worker for progression and development are not recognised |
| Competent in cross-cultural work relevant to your local community | 1 | 1 | 1 | Lack of awareness of cross-cultural issues, not connecting with people in an appropriate and respectful way |
| Stimulating and diverse work from day to day utilising a range of skills and approaches | 2 | 2 | 1 | Routine work |
| Motivated by the impact that you can have on someone that you are working with- that you can make a difference | 3 | 1 | 1 | Feeling that your work doesn't make a difference and therefore motivated by something else e.g. career path/pay etc. |
| Feel a sense of belonging to the community because you live there and therefore you know the context of your patients/clients | 5 | 5 | 1 | Patients are viewed as patients/client rather than as community members (you don't know their context) |
| Professional has multiple roles in relationships and so has to be able to deal with this | 5 | 5 | 1 | Professional is anonymous |

## Repertory Grid K

| \| **Personal/motivation qualities that contribute to successful practice in the job**  **1** \| \| --- \| | **Myself as I am currently** | **Myself in my ideal job** | **Most successful remote practitioner** | **5** |
| --- | --- | --- | --- | --- | --- |
| **Construct (similarity pole)** | Ratings from 1 on the left to 5 on the right | | | **Construct (contrast pole)** |
| Client-centred looking for best discharge not just getting them out; doctors were supportive | 1 | 1 | 2 | Time pressure is there; need to be efficient and doctors were keen for discharge due to bed pressure |
| Limited access to resources to meet client needs but good at networking for support and information to fill that gap | 2 | 4 | 3 | Having easy access to clinical specialists (or only having a single focus for your caseload), equipment, therapy tools etc. |
| We 'do everything' but don't specialise in anything- you have to know a little about everything- Jack of all trades/master of none | 1 | 3 | 3 | Opportunity to specialise in an area- developing expertise and knowledge in that area and can focus your PD in that area to a lot of depth |
| Hard to be confident that you know enough and feeling like you are only 'getting by' (you don't have anyone to compare with to check) | 2 | 4 | 4 | Confident that you know your area very well because you work in it every day and know how to deal with it |
| Value being challenged and like variety in patients and work location | 1 | 3 | 2 | Value being recognised as a specialist in an area and doing the same thing is not boring |
| There is no-one to refer to, so you deal with all aspects of patient care and patients get to know you better and you understand the patient's whole journey from sickness through recovery | 1 | 3 | 2 | Patients can be discharged and referred to another service; but handover can create gaps for the patient and information can get lost in handover |
| Patients are appreciative of service and are willing to help themselves | 1 | 1 | 2 | Patients act like the world owes them and they need 'it' yesterday |
| Efficiency of service delivery and numbers of patients seen are less important than providing a service | 1 | 1 | 1 | Efficiency of service is important- targets are set for occasions of service, geographical limits on provision of home visits |

## Repertory Grid N

| \| **Personal/motivation qualities that contribute to successful practice in the job**  **1** \| \| --- \| | **Myself as I am currently** | **Myself in my ideal job** | **Most successful remote practitioner** | **5** |
| --- | --- | --- | --- | --- | --- |
| **Construct (**similarity pole) | Ratings from 1 on the left to 5 on the right | | | **Construct** (contrast pole) |
| Confident in ability to steer the client but allow them to call the shots- a holistic approach or family-centred practice (experience is key) | 2 | 1 | 1 | (Self) focussed on the therapy process and outcome rather than the impact on the family and client |
| Ability to manage time and tasks efficiently to prevent feeling overwhelmed | 1 | 1 | 1 | Overwhelmed by daily job demands- phone calls, paperwork, client contact, planning for trips |
| Skilful at planning for travel and including contingency planning for DNAs, creative with resource selection and adaptable with resources, assessment choices | 1 | 1 | 1 | Slow and less skilful at planning for travel |
| Engaging with the community (talks, client-based discussions with daycare, doctor/teacher;) local community knew you as a person- you were approachable and trusted as both a health professional and a community member | 1 | 1 | 1 | Kept within the 4 walls and responsible to your patient and their immediate family; trusted as a health professional rather than as a person |
| Broad range of clients results in enjoyment and skill development | 1 | 1 | 1 | Lack of variety in clients creates boredom and narrowing of skills |
| Involvement with the community, inside and outside of work is professionally and personally fulfilling - (has influenced my retention) | 1 | 1 | 2 | 9-5 job where your personal life is separate from your professional life, (different circles of friends) |
| Sense of adventure and new possibilities was the catalyst for starting a rural career | 2 | 1 | 2 | Being in your comfort zone; not wanting to strike out on your own; comfortable with same old/same old; rejuvenate yourself through changing jobs rather than geographical location |
| Flexible in applying policies i.e. using your discretion for caseload management | 1 | 1 | 1 | Sticking to departmental policies for caseload management |
| Small size of community facilitates easier communication between professionals working with families across agencies for the families benefit; this is driven by the professionals | 1 | 1 | 1 | Communication between professionals is more difficult because you might not know the referrer etc., and families can slip through the gaps |
| Accounting for how the specifics of culture or the local 'cycles' impact on your work | 2 | 1 | 2 | Aware that culture or local 'cycles' have impact on clients but not really taking these into account |
| Self-motivating; to keep improving the way you work, or improving your department; you are left to your own devices and responsible for your own quality of work- you are trusted to do a good job and this provides satisfaction | 1 | 1 | 1 | Work is directed by a manager rather than being self-directed; management style is more controlling |
